# Copper-Coordinated Hyaluronic Acid Hydrogels with Antibacterial and Anti-Inflammatory Activities

**DOI:** 10.3390/molecules31132368

**Published:** 2026-07-05

**Authors:** Jiajie Chen, Haotian Huang, Yihan Wang, Ran Cheng, Wei Chen, Yanru Liu, Xiaobing Chen, Dongsheng Yang

**Affiliations:** 1 School of Life Science, Zhuhai College of Science and Technology, Zhuhai 519041, China; lf701429asd@163.com (J.C.); 15875664659@163.com (H.H.); w18904411011@outlook.com (Y.W.); chengran24@jlu.edu.cn (R.C.); missliuyr@outlook.com (Y.L.); 2 College of Life Science, Jilin University, Changchun 130012, China

**Keywords:** hydrogel dressings, hyaluronic acid, copper ions, curcumin micelles, antibacterial, anti-inflammatory, antioxidant

## Abstract

Chronic infected wounds are often characterized by persistent bacterial colonization, biofilm formation, excessive oxidative stress, and prolonged inflammation, which severely impair tissue regeneration. To address these challenges, a multifunctional wound dressing capable of antibacterial activity and microenvironment modulation was developed. In this study, amide-modified hyaluronic acid (HA-ADH) was used as the matrix, and a dynamic coordination network was constructed via Cu^2+^-hydrazide interactions to form an in situ HA-Cu hydrogel. Curcumin-loaded DSPE-PEG2000 micelles were further incorporated to obtain a pH-responsive composite hydrogel (HA-Cu/Cur). The prepared hydrogel exhibited a porous interconnected structure, along with favorable injectability, self-healing capability, tissue adhesiveness, moderate swelling, controllable degradability, and pH-responsive behavior under acidic conditions. In vitro antibacterial assays demonstrated that both HA-Cu and HA-Cu/Cur effectively inhibited the growth and biofilm formation of *Escherichia coli* and Staphylococcus aureus. The antibacterial activity was associated with disruption of bacterial morphology, depletion of intracellular ATP, and induction of reactive oxygen species, while HA-Cu/Cur showed enhanced performance in antibiofilm activity and oxidative stress-related effects compared with HA-Cu. Cytocompatibility studies revealed that the hydrogel extracts exhibited negligible cytotoxicity toward L929 fibroblasts and RAW 264.7 macrophages, while promoting fibroblast migration and significantly reducing the expression of pro-inflammatory cytokines (TNF-α, IL-6, and IL-1β) in lipopolysaccharide-stimulated RAW 264.7 cells, with HA-Cu/Cur showing a more pronounced anti-inflammatory effect. In summary, the HA-Cu/Cur hydrogel integrates the antibacterial and pro-healing properties of Cu^2+^ with the antioxidant and anti-inflammatory activities of curcumin. The hydrogel effectively inhibited the growth and biofilm formation of both *E. coli* and *S. aureus*, reduced the expression of TNF-α, IL-6, and IL-1β in LPS-stimulated macrophages, and promoted fibroblast migration, demonstrating its potential as a multifunctional wound dressing for the management of infected wounds.

## 1. Introduction

Cutaneous wound healing is a highly coordinated and dynamic biological process that generally proceeds through the phases of hemostasis, inflammation, proliferation, and remodeling, and depends on the orderly progression of multiple events, including immune regulation, cell migration, extracellular matrix remodeling, and angiogenesis [1,2]. However, in chronic wounds such as diabetic foot ulcers, pressure ulcers, and infected traumatic wounds, this process is frequently disrupted by persistent infection, dysregulated inflammation, and oxidative stress [3]. As a result, the wound remains trapped in a pathological state and fails to progress efficiently into the proliferative and remodeling phases, ultimately leading to delayed or non-healing wounds and imposing a substantial burden on both patients and healthcare systems [4].

In the microenvironment of chronic and infected wounds, bacterial colonization and biofilm formation are recognized as major barriers to healing [5]. Biofilms, which are formed by bacterial cells embedded in self-secreted extracellular polymeric substances, not only hinder the penetration of antimicrobial agents but also enable bacteria to evade immune clearance, thereby driving a vicious cycle of recurrent infection and persistent inflammation [6]. In addition, sustained elevation of pro-inflammatory cytokines and excessive accumulation of reactive oxygen species (ROS) further damage cellular membranes, proteins, and nucleic acids, while impairing fibroblast migration, collagen deposition, and angiogenesis [7,8]. These pathological changes aggravate microenvironmental imbalance and delay tissue repair. Therefore, for chronic and infected wounds, strategies based solely on antibacterial treatment or passive wound coverage are often insufficient to simultaneously address infection control, inflammation resolution, and oxidative stress regulation.

At present, conventional debridement and antibiotic therapy can reduce bacterial burden to some extent, but their effectiveness against biofilm-associated infections remains limited [9]. Debridement alone cannot prevent the rapid re-establishment of biofilms, whereas antibiotic penetration and bactericidal efficacy are compromised by the extracellular matrix barrier and the presence of tolerant bacterial populations, which may also increase the risk of antimicrobial resistance [10]. Traditional passive dressings such as gauze mainly serve as absorbent and protective coverings and lack the ability to actively modulate the pathological wound microenvironment. In contrast, bioactive dressings with drug-delivery and microenvironment-regulating functions, especially multifunctional hydrogel systems, have attracted increasing attention in recent years [11]. Owing to their high water content, good biocompatibility, tunable structure, and capacity for loading bioactive agents, hydrogels can maintain a moist wound environment while providing integrated antibacterial, anti-inflammatory, antioxidant, and pro-regenerative effects through rational functional design [12,13].

Hyaluronic acid (HA), a major natural polysaccharide in the extracellular matrix, has been widely used in wound dressings and tissue engineering materials because of its excellent biocompatibility, biodegradability, and moisture-retention capability, as well as its involvement in cell adhesion, migration, and tissue repair [14]. However, native HA has limited intrinsic antibacterial activity, and conventional HA hydrogels often fail to simultaneously achieve the injectability, self-healing ability, and environmental responsiveness required for the treatment of complex wounds [15]. Therefore, functional modification of HA and construction of dynamic crosslinked networks represent effective strategies for endowing HA-based hydrogels with enhanced structural and biological functions [16,17].

Among the various approaches for hydrogel design, metal ion-crosslinked hydrogels have emerged as a promising platform because they integrate structural functionality with biological activity. Copper ions (Cu^2+^), in particular, can serve not only as dynamic crosslinking nodes for hydrogel formation but also as bioactive components with broad-spectrum antibacterial properties and multiple roles in tissue repair [18]. On the one hand, Cu^2+^ can inhibit bacteria and biofilms by disrupting membrane integrity, interfering with metabolic processes, and inducing oxidative stress [19]. On the other hand, as a cofactor for a variety of redox- and matrix-related enzymes, Cu^2+^ is closely associated with angiogenesis, extracellular matrix remodeling, and tissue regeneration [20]. Compared with some metal-based factors that mainly provide antibacterial activity alone, Cu^2+^ is therefore a more attractive functional component for simultaneously controlling infection and promoting wound healing.

In addition to metal ions, natural bioactive molecules are also important candidates for regulating the wound microenvironment [21]. Curcumin (Cur), a natural polyphenol, possesses well-documented anti-inflammatory, antioxidant, and moderate antibacterial activities, and can alleviate oxidative stress and persistent inflammation in chronic wounds by scavenging free radicals and modulating inflammation-related signaling pathways [22]. Nevertheless, its poor water solubility, limited stability, and low bioavailability substantially restrict its application in aqueous systems and local delivery [23]. The use of nanomicellar delivery systems to improve its dispersibility and local availability is therefore considered a feasible strategy for enhancing its therapeutic potential [24].

Although various HA-based hydrogels, metal ion-crosslinked systems, and curcumin-containing wound dressings have been reported, several limitations remain. Many antibacterial hydrogels primarily focus on infection control while lacking sufficient anti-inflammatory functionality, whereas curcumin-loaded systems often suffer from poor structural stability or inadequate antibacterial efficacy [25,26]. In addition, relatively few studies have integrated dynamic Cu^2+^-coordination crosslinking with nanomicelle-mediated curcumin delivery in a single hydrogel platform capable of simultaneously addressing bacterial infection, biofilm formation, inflammation, and oxidative stress. Therefore, the development of a multifunctional hydrogel system that combines structural adaptability with synergistic antibacterial and anti-inflammatory activities remains highly desirable.

Based on these considerations, we proposed a metal coordination-driven strategy for synergistic wound microenvironment modulation. In this design, adipic dihydrazide-modified hyaluronic acid (HA-ADH) was used as the matrix, and a pH-responsive hydrogel network was formed in situ under mild conditions through dynamic coordination between Cu^2+^ and hydrazide-related binding sites. Meanwhile, curcumin was encapsulated into DSPE-PEG_2000_ micelles and incorporated into the hydrogel system to fabricate a multifunctional HA-Cu/Cur hydrogel. This system was designed to combine the biocompatibility and biomimetic matrix properties of HA, the antibacterial and pro-healing functions of Cu^2+^, and the anti-inflammatory and antioxidant activities of Cur, thereby enabling coordinated regulation of bacterial growth, biofilm formation, oxidative stress, and inflammatory responses in infected wounds. To the best of our knowledge, this study is among the few reports employing Cu^2+^-mediated dynamic coordination together with curcumin-loaded micelles in a hyaluronic acid hydrogel system. We hypothesized that integrating the antibacterial activity of Cu^2+^ with the anti-inflammatory and antioxidant properties of curcumin within a dynamically coordinated HA hydrogel would synergistically regulate bacterial infection, biofilm formation, oxidative stress, and inflammatory responses. Therefore, a multifunctional HA-Cu/Cur hydrogel was developed and systematically evaluated for its physicochemical properties, antibacterial performance, biocompatibility, and anti-inflammatory activity.

## 2. Results and Discussion

### 2.1. Preparation and Characterization of Hydrogels

**Figure 1:**
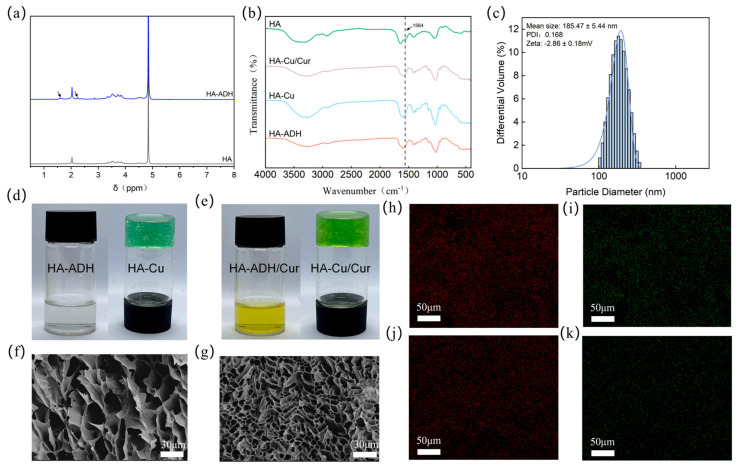
Structural characterization of HA-ADH and Cur, and morphological characterization of HA-Cu and HA-Cu/Cur hydrogels. (**a**) 1H NMR spectrum of HA-ADH. (**b**) FTIR spectra of HA-ADH. (**c**) Particle size distribution of Cur micelles. (**d**,**e**) Representative photographs of HA-Cu and HA-Cu/Cur hydrogels. (**f**,**g**) SEM images of the freeze-dried HA-Cu and HA-Cu/Cur hydrogels. (**h**,**i**) EDS elemental mapping of Cu and N in HA-Cu hydrogel. (**j**,**k**) EDS elemental mapping of Cu and N in HA-Cu/Cur hydrogel.

The successful grafting of adipic dihydrazide (ADH) onto the HA backbone was confirmed by both 1H NMR and FTIR analyses. As shown in Figure 1a, new characteristic signals appeared at 1.55–1.72 ppm and 2.18–2.40 ppm, which were assigned to the methylene protons of the ADH chain, confirming the successful introduction of ADH with a substitution degree of 20% [27]. Further quantitative analysis based on the integral ratio of the characteristic ADH signals to the N-acetyl methyl peak indicated that the degree of substitution (DS) of ADH was approximately 20%. FTIR spectra further supported the formation of HA-ADH (Figure 1b). Compared with native HA, HA-ADH retained the characteristic absorption bands of HA while exhibiting a new amide II vibration band at 1564 cm^−1^, which was attributed to the hydrazide group [28]. In both HA-Cu and HA-Cu/Cur hydrogels, the characteristic amide-related vibration bands were retained. Changes in the amide absorption region after Cu^2+^ incorporation, together with the successful formation of stable hydrogels, were consistent with the occurrence of coordination interactions between Cu^2+^ and hydrazide groups [29]. No additional vibration bands associated with new covalent bond formation were observed after Cur incorporation, indicating that Cur was physically loaded into the hydrogel network rather than chemically conjugated.

Cur micelles were prepared by the thin-film hydration method, in which DSPE-PEG_2000_ formed a core–shell nanostructure to improve the dispersion and solubility of Cur in aqueous media. As shown in Figure 1c, dynamic light scattering (DLS) analysis showed that the Cur micelles had an average hydrodynamic diameter of 185.47 ± 5.44 nm, a polydispersity index (PDI) of 0.168 ± 0.11, and a zeta potential of −2.86 ± 0.18 mV. These results indicate a relatively narrow size distribution and good colloidal uniformity, although the low absolute zeta potential suggests limited electrostatic stabilization. This behavior is likely compensated by steric stabilization provided by the PEG corona, which is typical for DSPE-PEG-based micellar systems [30]. In addition, the encapsulation efficiency and drug loading of Cur reached 88.49 ± 1.36% and 0.88 ± 0.12%, respectively, indicating that Cur was effectively entrapped within the hydrophobic core of the micelles. The micellar formulation is expected to improve the dispersibility and bioavailability of Cur in aqueous environments, which may contribute to the observed antioxidant and anti-inflammatory activities of the hydrogel system.

As shown in Figure 1d,e, both HA-Cu and HA-Cu/Cur formed intact and stable hydrogels without obvious flow upon vial inversion, indicating that the Cu^2+^-mediated coordination network could be rapidly established under mild conditions and endowed the system with good structural integrity. HA-Cu appeared as a semi-transparent light green hydrogel, whereas HA-Cu/Cur exhibited a brighter yellow-green color after Cur incorporation. The homogeneous appearance of HA-Cu/Cur suggested that Cur was well dispersed in the hydrogel matrix and did not disrupt gel formation.

As shown in Figure 1f,g, SEM observation of the freeze-dried cross-sections revealed that both hydrogels possessed a typical sponge-like three-dimensional porous structure. Such an interconnected porous morphology is favorable for fluid absorption, oxygen and nutrient transport, and may provide space for subsequent cell adhesion and tissue ingrowth [31]. HA-Cu (Figure 1f) exhibited relatively larger pores with smoother pore walls and good interconnectivity, which may facilitate liquid uptake and solute diffusion. In contrast, HA-Cu/Cur (Figure 1g) displayed smaller pores and rougher pore walls, suggesting that the incorporation of Cur micelles altered the microstructural organization of the coordination network and rendered the internal structure more compact [32]. This structural change may contribute to improved network stability and potentially enhance the retention of active components and metal ions within the hydrogel [33,34].

As shown by EDS elemental mapping (Figure 1h–k, both Cu and N were uniformly distributed throughout the two hydrogel matrices, with no obvious local aggregation. This result indicates that Cu^2+^ participated homogeneously in the coordination crosslinking of the HA-ADH network and further supports the structural uniformity of the resulting hydrogels. Taken together, the macroscopic appearance, microscopic morphology, and elemental distribution demonstrate that both HA-Cu and HA-Cu/Cur could be successfully formed with relatively homogeneous internal architectures, thereby providing a structural basis for their subsequent mechanical performance and biological functions.

### 2.2. Swelling, Degradation, Antioxidant, and Rheological Properties of Hydrogels

**Figure 2:**
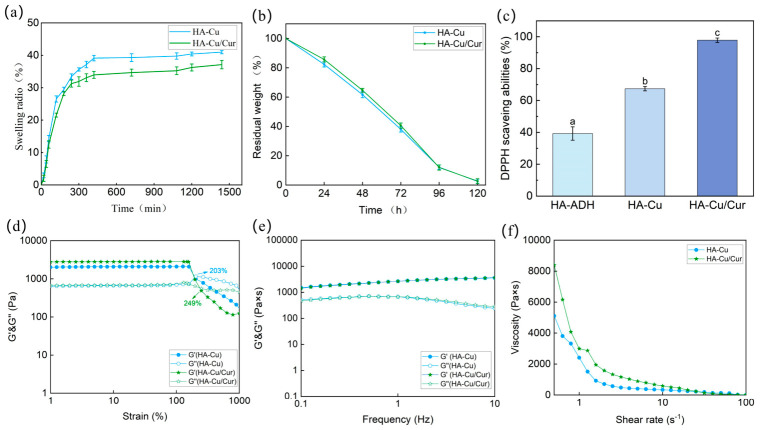
Swelling, degradation, antioxidant, and rheological characterization of the hydrogels. (**a**) Swelling ratio of the hydrogels. (**b**) Degradation profile of the hydrogels. (**c**) DPPH radical scavenging activity of the hydrogels. (**d**) Strain sweep curves of the hydrogels. If the lowercase letters are not the same, it indicates a significant difference betwween groups, *p* < 0.05, and vice versa. (**e**) Frequency sweep curves of the hydrogels. (**f**) Viscosity as a function of shear rate for the hydrogels.

Swelling behavior is an important parameter for wound dressing applications. As shown in Figure 2a, both HA-Cu and HA-Cu/Cur hydrogels exhibited rapid swelling in PBS and reached equilibrium within 300 min. The equilibrium swelling ratio of HA-Cu was 40.97 ± 0.50%, whereas that of HA-Cu/Cur was slightly lower at 37.13 ± 1.31%, indicating that the incorporation of Cur slightly reduced the swelling capacity of the hydrogel. This decrease may be associated with the incorporation of Cur-loaded micelles, which acted as physical fillers within the hydrogel network and increased the compactness of the internal structure. Such a structural feature may restrict water uptake and contribute to the enhanced structural stability of the hydrogel [35].

The degradation profiles shown in Figure 2b indicate that both HA-Cu and HA-Cu/Cur gradually lost mass over time and were almost completely degraded within 120 h. This degradation behavior is consistent with that generally reported for HA-based materials, which can undergo degradation through hydrolytic, enzymatic, and oxidative pathways [36]. Although the incorporation of Cur slightly slowed the degradation process, no statistically significant difference was observed between the HA-Cu and HA-Cu/Cur groups (*p* > 0.05), suggesting that Cur had no substantial effect on the overall degradation rate of the hydrogels.

The antioxidant activity evaluated by the DPPH radical scavenging assay (Figure 2c) showed that the scavenging rates of HA-ADH, HA-Cu, and HA-Cu/Cur were 39.26 ± 4.19%, 67.37 ± 1.34%, and 97.85 ± 1.35%, respectively, with significant differences observed among the groups (*p* < 0.05). Among them, HA-Cu/Cur exhibited the highest free radical scavenging capacity, indicating that Cur loading markedly enhanced the antioxidant activity of the system. This improvement is beneficial for alleviating oxidative stress-related microenvironmental imbalance in chronic and infected wounds.

Rheological characterization was further performed to evaluate the structural stability and injectability of the hydrogels. As shown in Figure 2d, strain amplitude sweep tests revealed that both hydrogels displayed typical gel-like behavior at low strain, with G′ consistently higher than G″, indicating that the Cu^2+^–hydrazide coordination network remained stable under small deformation. With increasing strain, G′ decreased and approached G″, suggesting progressive disruption of the network, which is characteristic of dynamically crosslinked hydrogels. The critical strain values of HA-Cu and HA-Cu/Cur were approximately 203% and 249%, respectively, indicating that Cur incorporation improved the deformation tolerance of the hydrogel without compromising its elasticity. In addition, the storage modulus (G′) of HA-Cu/Cur remained higher than that of HA-Cu throughout the tested strain range, suggesting enhanced network integrity and mechanical strength. This enhancement may be attributed to the incorporation of Cur-loaded micelles, which did not disrupt the Cu^2+^–hydrazide coordination network but instead acted as physical fillers within the hydrogel matrix [37]. The presence of these micelles may increase physical chain entanglement and introduce spatial confinement effects, thereby restricting polymer chain mobility and improving the overall viscoelastic properties of the hydrogel [38]. As shown in Figure 2e, both hydrogels maintained G′ > G″ over the entire frequency range, further confirming their stable gel characteristics.

As shown in Figure 2f, both hydrogels exhibited pronounced shear-thinning behavior, with viscosity decreasing as the shear rate increased, indicating that the materials could readily flow under external forces such as injection or spreading. HA-Cu/Cur showed higher viscosity than HA-Cu, particularly at low shear rates, reflecting its more compact crosslinked network. Such a higher viscosity under static conditions may contribute to better retention and stability on the wound surface, while the preserved shear-thinning behavior remains favorable for practical application [39].

### 2.3. pH Responsiveness, Adhesion, Self-Healing, and Injectability of Hydrogels

**Figure 3:**
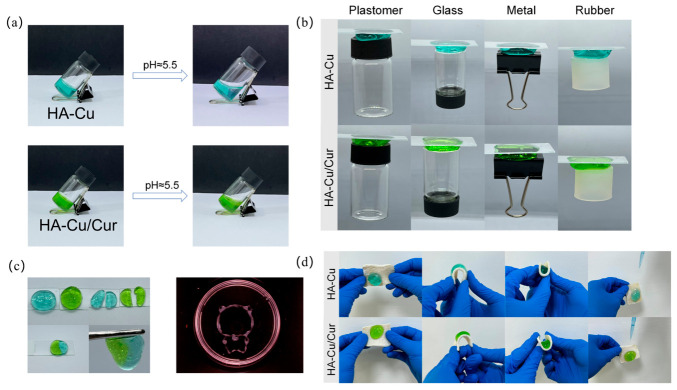
Characterization of the pH responsiveness, adhesion, self-healing, and injectability of the hydrogels. (**a**) Visual evaluation of the pH-responsive behavior of the hydrogels. (**b**,**d**) Adhesive properties of the hydrogels on biological and non-biological substrates. (**c**) Self-healing behavior and injectability of the hydrogels.

As shown in Figure 3a, both HA-Cu and HA-Cu/Cur maintained their hydrogel shape under neutral conditions but became loosened and contracted at approximately pH 5.5, indicating clear pH-responsive behavior. This phenomenon suggests the potential of these hydrogels for responsive release in infected wounds, where local acidification commonly occurs as a result of bacterial metabolism and inflammatory activity. Under such conditions, network relaxation may facilitate the release of Cu^2+^ and Cur from the hydrogel matrix.

The self-healing ability of the hydrogels is shown in Figure 3c. After being cut and brought back into contact for 30 min without any external stimulus, the hydrogel pieces rejoined into an integral construct, demonstrating effective self-healing behavior. This property is likely attributable to the dynamic reversibility of the Cu^2+^–hydrazide coordination interactions, which enables reconstruction of the network after damage. In addition, extrusion of the hydrogel through a 22G needle into a bear-shaped pattern further demonstrated its injectability and structural recovery capacity, indicating its suitability for applications requiring both deformability during administration and shape retention after placement.

Adhesive performance is essential for wound dressings because it helps prevent detachment during patient movement and minimizes relative displacement at the wound interface. As shown in Figure 3b, both HA-Cu and HA-Cu/Cur hydrogels adhered stably to hydrated porcine skin, which was used to simulate a moist tissue environment. During bending, rolling, and inversion, both hydrogels remained firmly attached without obvious sliding, cracking, or edge lifting. The addition of PBS to the surface did not cause detachment or blistering, further indicating stable adhesion on soft and hydrated biological substrates. This property is advantageous for sustained wound coverage and localized drug delivery during the healing process. As shown in Figure 3d, both hydrogels also exhibited stable adhesion on a variety of substrates, including plastic, glass, metal, and rubber, without obvious slipping or detachment. These results suggest that the incorporation of Cur did not compromise the adhesive performance of the material.

The adhesion likely arises from the combined effects of interfacial wetting by the HA backbone and the structural stability provided by Cu^2+^-mediated coordination within the hydrogel network. Specifically, the abundant hydroxyl (−OH), carboxylate (−COO^−^), and amide/hydrazide groups present in the hydrogel can form hydrogen-bonding interactions with biological tissues and substrate surfaces [40]. In addition, carboxylate groups may participate in electrostatic and coordination interactions with surface metal ions or positively charged sites, while the hydrated polymer chains facilitate intimate interfacial contact through efficient surface wetting [41]. These combined interactions contribute to the observed adhesive behavior on diverse substrates.

### 2.4. Antibacterial Activity of Hydrogels

**Figure 4:**
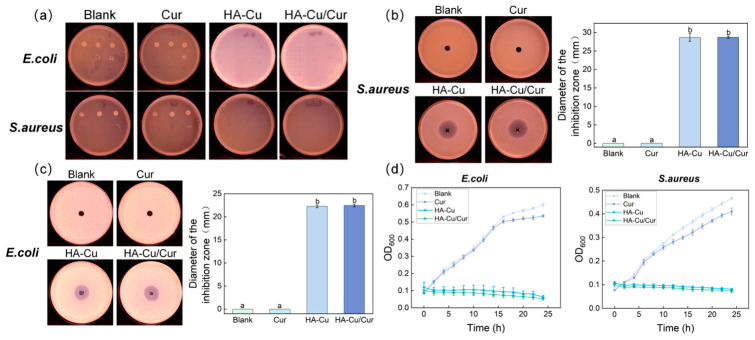
Antibacterial activity of the hydrogels. (**a**) Representative colony images from plate counting assays of bacteria treated with PBS, Cur, HA-Cu, and HA-Cu/Cur. (**b**) Representative photographs and quantitative analysis of inhibition zones against *S. aureus* after treatment with PBS, Cur, HA-Cu, and HA-Cu/Cur. (**c**) Representative photographs and quantitative analysis of inhibition zones against *E. coli* after treatment with PBS, Cur, HA-Cu, and HA-Cu/Cur. (**d**) Growth curves of *E. coli* and *S. aureus* after treatment with PBS, Cur, HA-Cu, and HA-Cu/Cur. If the lowercase letters are not the same, it indicates a significant difference betwween groups, *p* < 0.05, and vice versa.

The antibacterial activity of the hydrogels against *E. coli* and *S. aureus* was first evaluated by colony counting. As shown in Figure 4a, abundant bacterial colonies were observed in the Blank group in both undiluted and diluted samples, indicating active bacterial proliferation. The Cur group showed a reduction in colony number for both bacterial strains, suggesting a certain degree of antibacterial activity; however, under the tested concentration and incubation time, Cur alone was insufficient to completely inhibit bacterial growth. In contrast, both HA-Cu and HA-Cu/Cur exhibited pronounced antibacterial effects, with no visible colonies observed in either undiluted or diluted samples, indicating that the bacteria were almost completely eliminated. These results demonstrate that both hydrogels possess strong antibacterial activity and that the incorporation of Cur did not compromise the antibacterial efficacy of Cu^2+^.

The inhibition zone assay, a standard method for evaluating localized antibacterial activity, further confirmed these findings. As shown in Figure 4b,c, no visible inhibition zones were observed in the Blank or Cur groups, indicating that Cur alone could not establish an effective antibacterial concentration gradient in the agar medium. In contrast, both HA-Cu and HA-Cu/Cur generated distinct inhibition zones (*p* < 0.05). For *E. coli*, the inhibition zone diameters were 22.28 ± 0.28 mm and 28.66 ± 1.05 mm for HA-Cu and HA-Cu/Cur, respectively; for *S. aureus*, the corresponding values were 22.46 ± 0.30 mm and 28.71 ± 0.31 mm. Although no significant difference was observed between HA-Cu and HA-Cu/Cur, these results suggest that Cur may play an auxiliary role in enhancing antibacterial performance, whereas the potent antibacterial activity of the hydrogels is primarily attributable to the antibacterial effect of Cu^2+^ within the coordination network. Under acidic conditions associated with bacterial metabolism, weakening of the hydrazide–Cu^2+^ coordination interactions may induce partial relaxation of the hydrogel network, which could enhance the interaction between the hydrogel and the local wound microenvironment.

To further assess bacterial growth throughout the culture period, *E. coli* and *S. aureus* were treated with different samples and monitored by OD_600_ measurements. As shown in Figure 4d, the Cur group exhibited slightly lower OD_600_ values than the Blank group, indicating a modest inhibitory effect on bacterial growth. In contrast, both HA-Cu and HA-Cu/Cur maintained consistently low OD_600_ values throughout the entire culture period, and no obvious bacterial proliferation was observed. These results indicate that both hydrogels exert sustained antibacterial activity, further supporting their potential application in wound healing and infection management.

### 2.5. Antibacterial Mechanism of Hydrogels

**Figure 5:**
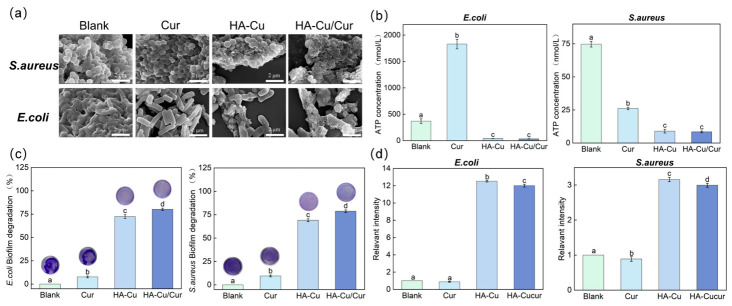
Antibacterial mechanism analysis of the hydrogels. (**a**) SEM images of *E. coli* and *S. aureus* after different treatments. (Scale bar: 2 μm) (**b**) Intracellular ATP levels of *E. coli* and *S. aureus* after treatment with PBS, Cur, HA-Cu, and HA-Cu/Cur. (**c**) Biofilm removal rates of *E. coli* and *S. aureus* after treatment with PBS, Cur, HA-Cu, and HA-Cu/Cur. (**d**) Relative fluorescence intensity of *E. coli* and *S. aureus* after different treatments, with the PBS-treated control group normalized to 1. If the lowercase letters are not the same, it indicates a significant difference betwween groups, *p* < 0.05, and vice versa.

To investigate the antibacterial mechanism of the materials, the morphological changes in *E. coli* and *S. aureus* after co-incubation with different samples were observed by SEM. As shown in Figure 5a, bacteria in the Blank group displayed typical morphology, with *S. aureus* appearing as clustered cocci and *E. coli* retaining a regular rod-like shape, indicating normal physiological status. After Cur treatment, *S. aureus* exhibited slight surface collapse, whereas *E. coli* showed bending and surface bulging, suggesting that Cur may disturb membrane integrity to some extent [42]. In the HA-Cu group, *S. aureus* displayed marked membrane damage, collapse, and fragmentation, while *E. coli* exhibited ruptured cell walls and leakage of intracellular contents. These observations suggest that the released Cu^2+^ interacted with negatively charged components on the bacterial surface, thereby disrupting membrane integrity and impairing essential cellular functions [43]. In the HA-Cu/Cur group, membrane damage was even more severe, and most cells appeared as irregular fragments or severely collapsed structures, indicating that the combination of Cur and Cu^2+^ enhanced the bactericidal effect.

Because biofilm formation is a key feature of chronic wound infection, biofilm removal was quantitatively evaluated by crystal violet staining. As shown in Figure 5c, the Cur group exhibited relatively low biofilm removal rates, reaching only 7.67 ± 1.00% for *E. coli* and 9.50 ± 1.01% for *S. aureus*, indicating that Cur alone had limited antibiofilm activity at the tested concentration. In contrast, HA-Cu significantly improved biofilm removal, with removal rates of 72.44 ± 1.98% for *E. coli* and 69.00 ± 1.53% for *S. aureus*, which was mainly attributed to the antibacterial action of Cu^2+^. Notably, HA-Cu/Cur further enhanced biofilm removal to 80.19 ± 1.27% for *E. coli* and 78.71 ± 1.44% for *S. aureus*. These data support a synergistic contribution of Cur and Cu^2+^ in biofilm disruption, possibly because Cur facilitates membrane perturbation and improves the susceptibility of biofilm-embedded bacteria to Cu^2+^.

Intracellular ATP levels were measured as an indicator of bacterial metabolic activity and viability. As shown in Figure 5b, both *E. coli* and *S. aureus* in the Blank group maintained relatively high ATP levels, indicating normal metabolic activity. In the Cur group, the ATP level of *S. aureus* decreased to 26.12 ± 0.92 nmol/L, suggesting inhibition of energy metabolism. In contrast, the ATP level of *E. coli* increased to 1830.14 ± 90.76 nmol/L, which may reflect stress-induced metabolic reprogramming [42]. In both the HA-Cu and HA-Cu/Cur groups, intracellular ATP levels in *E. coli* and *S. aureus* were significantly reduced (*p* < 0.05), indicating that the hydrogels effectively suppressed bacterial energy metabolism, which may be related to Cu^2+^-mediated interference with electron transport-associated processes and oxidative stress induction [44].

To further evaluate the involvement of oxidative stress, intracellular ROS levels in *E. coli* and *S. aureus* were detected using the DCFH-DA probe. As shown in Figure 5d, the Cur group exhibited relatively low ROS levels compared with the Blank group (*E. coli*, 0.88 ± 0.05; *S. aureus*, 0.89 ± 0.07), consistent with the intrinsic antioxidant property of Cur [45]. In the HA-Cu group, ROS levels increased markedly (*E. coli*, 12.52 ± 0.09; *S. aureus*, 3.16 ± 0.06), indicating that Cu^2+^ was associated with pronounced oxidative stress in bacterial cells. Notably, the HA-Cu/Cur group also maintained substantially elevated ROS levels (*E. coli*, 12.01 ± 0.05; *S. aureus*, 3.00 ± 0.04), comparable to those observed in the HA-Cu group. These results suggest that the incorporation of Cur did not substantially impair the ROS-associated antibacterial activity of Cu^2+^. Combined with the ATP depletion, bacterial membrane damage, and enhanced biofilm removal observed in the HA-Cu/Cur group, the antibacterial effect of the composite hydrogel is likely attributable to the synergistic contribution of multiple mechanisms rather than excessive ROS generation alone.

Taken together, the antibacterial activity of HA-Cu and HA-Cu/Cur hydrogels appears to be driven by three main factors: disruption of bacterial membrane integrity, suppression of cellular energy metabolism, and inhibition/removal of biofilms. The antibacterial activity of the hydrogels was closely associated with the presence of Cu^2+^, as evidenced by the marked reduction in intracellular ATP levels and the elevated ROS production observed in both *E. coli* and *S. aureus*. Meanwhile, Cur may enhance these effects mainly by increasing membrane vulnerability and improving antibiofilm performance. In particular, HA-Cu/Cur exhibited stronger effects on membrane damage and biofilm removal than HA-Cu alone, highlighting the functional advantage of incorporating Cur into the Cu^2+^-coordinated hydrogel system. These combined mechanisms endow the hydrogel with potent antibacterial performance and support its potential as a multifunctional material for wound healing and infection control.

### 2.6. Cellular Functional Evaluation of Hydrogels

**Figure 6:**
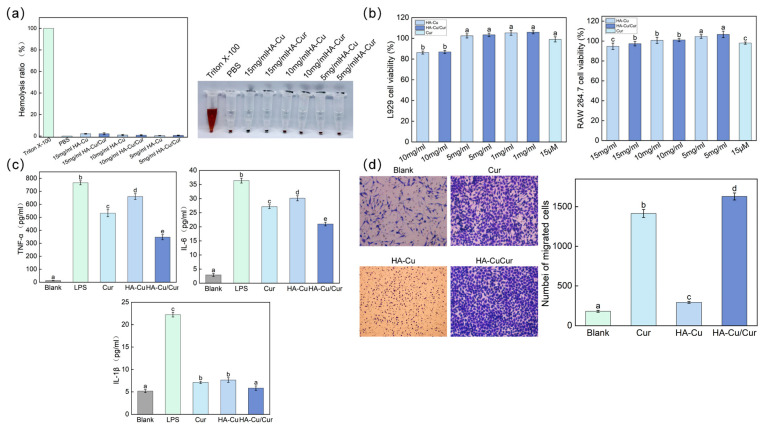
Cellular functional evaluation of the hydrogels. (**a**) Hemolytic effect of the hydrogels on red blood cells. (**b**) Effects of Cur, HA-Cu, and HA-Cu/Cur on the viability of RAW 264.7 cells and L929 cells. (**c**) Effects of Cur, HA-Cu, and HA-Cu/Cur on the Transwell migration of L929 cells. (**d**) Effects of Cur, HA-Cu, and HA-Cu/Cur on the secretion of inflammatory cytokines in RAW 264.7 cells: TNF-α, IL-6, and IL-1β. If the lowercase letters are not the same, it indicates a significant difference betwween groups, *p* < 0.05, and vice versa.

To assess the in vitro biosafety of HA-Cu and HA-Cu/Cur hydrogels as candidate wound dressing materials, their hemocompatibility and cytocompatibility were first investigated. As shown in Figure 6a, both hydrogel extracts exhibited very low hemolysis rates at concentrations of 15, 10, and 5 mg/mL. After centrifugation, the appearance of the supernatants was similar to that of the PBS negative control, whereas obvious hemolysis was observed in the Triton X-100 positive control group. These results indicate that neither material caused significant erythrocyte membrane damage under the experimental conditions, demonstrating good hemocompatibility.

On this basis, the cytocompatibility of the materials was further evaluated using L929 fibroblasts and RAW 264.7 macrophages. As shown in Figure 6b, after treatment with HA-Cu and HA-Cu/Cur hydrogel extracts over the tested concentration range, the viability of both cell types remained above 80%. Similarly, cells treated with 15 μM curcumin also maintained high viability, with survival rates of 98.99 ± 2.43% for L929 cells and 97.96 ± 1.14% for RAW 264.7 cells. These results indicate that the incorporation of Cur did not compromise the basic cytocompatibility of the system. Based on these findings, 5 mg/mL hydrogel extract and 15 μM Cur were selected as the working concentrations for the subsequent cell function studies.

After confirming the basic biosafety of the materials, their effects on fibroblast migration were further examined. As shown in Figure 6c, the Transwell migration assay demonstrated that Cur, HA-Cu, and HA-Cu/Cur all promoted L929 cell migration to different extents compared with the Blank group, although with clear differences in magnitude. The Cur group showed a significant increase in migrated cell number, and the HA-Cu group also exhibited a certain migration-promoting effect. Notably, the HA-Cu/Cur group produced the strongest effect, with the number of migrated cells reaching 1629.67 ± 44.73, which was markedly higher than that in the Cur and HA-Cu groups. These results suggest that the composite hydrogel is more favorable for promoting fibroblast migration, a process that is crucial for granulation tissue formation and re-epithelialization. This enhanced effect may be related to the combined contribution of Cur in alleviating oxidative stress and Cu^2+^ in modulating the extracellular microenvironment [20].

Given that excessive inflammation is a major obstacle to wound repair, the inflammation-regulating capability of the materials was further evaluated using an LPS-induced RAW 264.7 inflammatory model. As shown in Figure 6d, LPS stimulation increased the levels of TNF-α, IL-6, and IL-1β to 766.11 ± 15.55 ng/mL, 36.37 ± 0.86 ng/mL, and 22.21 ± 0.51 ng/mL, respectively, indicating successful establishment of the inflammatory model. Compared with the LPS group, both Cur and HA-Cu reduced the levels of these pro-inflammatory cytokines to some extent, suggesting that each of them possessed anti-inflammatory activity. In contrast, the HA-Cu/Cur group showed the most pronounced inhibitory effect, reducing the levels of TNF-α, IL-6, and IL-1β to 346.78 ± 19.73 ng/mL, 20.98 ± 0.64 ng/mL, and 5.84 ± 0.49 ng/mL, respectively, which was significantly superior to the effects of the single-component treatments (*p* < 0.05). These results indicate that the composite system more effectively attenuated the excessive inflammatory activation of macrophages, which may be attributed to the synergistic effects of the anti-inflammatory and antioxidant properties of Cur and the biological activity of Cu^2+^ within the hydrogel network [20,46].

## 3. Materials and Methods

### 3.1. Materials and Reagents

Hyaluronic acid (HA, molecular weight 200 kDa), adipic dihydrazide (ADH), copper(II) chloride dihydrate (CuCl_2_·2H_2_O), curcumin (Cur), and 1,2-distearoyl-sn-glycero-3-phosphoethanolamine-PEG_2000_ (DSPE-PEG_2000_) were purchased from Sigma-Aldrich (St. Louis, MO, USA). Dichloromethane, methanol, 1,1-diphenyl-2-picrylhydrazyl (DPPH), hydrochloric acid, crystal violet solution, MTT, and paraformaldehyde were purchased from Macklin Biochemical Co., Ltd. (Shanghai, China). Fetal bovine serum (FBS), phosphate-buffered saline (PBS), penicillin/streptomycin, and lipopolysaccharide (LPS) were obtained from Gibco (Waltham, MA, USA). 2′,7′-Dichlorodihydrofluorescein diacetate (DCFH-DA), mouse IL-1β ELISA kit, and mouse TNF-α ELISA kit were purchased from Beyotime Biotechnology Co., Ltd. (Shanghai, China). Mouse fibroblasts (L929) and mouse macrophages (RAW 264.7) were obtained from the Cell Bank of the Chinese Academy of Sciences (Shanghai, China).

### 3.2. Preparation and Characterization of Cur

Curcumin-loaded micelles (Cur) were prepared using a modified thin-film hydration method [30]. Briefly, curcumin (2.0 mg) and DSPE-PEG2000 (10.0 mg) were dissolved separately in methanol and chloroform, mixed at a volume ratio of 1:1, and sonicated for 5 min. The organic solvents were then removed under reduced pressure to form a thin film, which was hydrated with 5 mL of 0.9% NaCl solution at 60 °C for 30 min. The resulting dispersion was filtered through a 0.22 μm membrane and used for subsequent experiments.

After appropriate dilution and sonication, the hydrodynamic diameter and polydispersity index (PDI) of Cur were measured by dynamic light scattering (DLS), and the zeta potential was determined by electrophoretic light scattering (ELS). The standard curve of curcumin was established in ethanol and measured at 422 nm, giving a linear regression equation of y = 0.0302x + 0.0526 (R^2^ = 0.9992). The Cur dispersion was sonicated and centrifuged at 12,000 rpm for 10 min, and the content of free curcumin in the supernatant was determined. The encapsulation efficiency (EE) and drug loading (DL) of the prepared Cur micelles were subsequently determined and are presented in the Section 2.

(1) $$\mathrm{Encapsulation}\;\mathrm{efficiency}(\%)=\frac{{\mathrm{curcumin}}_{\mathrm{total}\;\mathrm{amount}}-{\mathrm{curcumin}}_{\mathrm{supernatant}}}{{\mathrm{curcumin}}_{\mathrm{total}\;\mathrm{amount}}}\times 100\%$$

(2) $$\mathrm{Drug}\;\mathrm{loading}\;(\%)=\frac{\mathrm{curcumin}}{{(\mathrm{DSPE}-\mathrm{PEG}}_{2000}+\mathrm{curcumin})}\times 100\%$$

### 3.3. Synthesis of HA-ADH and Preparation of Hydrogels

#### 3.3.1. Synthesis of HA-ADH

HA-ADH was synthesized with slight modifications based on a previously reported method [27]. Briefly, HA (1.0 g, 2.5 mmol of repeating disaccharide units) and ADH (5.0 mmol) were dissolved in 90 mL of deionized water. After adjusting the pH of the solution to 5.5, NHS (0.6 mmol) and EDC·HCl (0.5 mmol) were added, and the reaction was allowed to proceed at room temperature for 36 h. The reaction mixture was then dialyzed against deionized water using a dialysis membrane with a molecular weight cut-off of 3.5 kDa for 72 h, followed by lyophilization to obtain HA-ADH.

#### 3.3.2. Preparation of Hydrogels

For hydrogel preparation, a 4 wt% HA-ADH solution, a 0.5 M CuCl_2_ solution, and a 4 wt% HA-ADH/Cur solution containing 15 μM Cur were prepared. Then, 1.0 mL of HA-ADH solution or HA-ADH/Cur solution was mixed with a predetermined volume of CuCl_2_ solution at room temperature so that the molar ratio of hydrazide groups to Cu^2+^ was 1:1 (1:1 ratio was selected based on preliminary optimization). After vortex mixing, the mixtures were allowed to stand until gelation, yielding HA-Cu and HA-Cu/Cur hydrogels, respectively. Unless otherwise specified, this formulation was used in all subsequent experiments.

### 3.4. Characterization of Hydrogels

#### 3.4.1. Characterization of HA-ADH and Hydrogels

HA, HA-ADH, and lyophilized HA-Cu and HA-Cu/Cur hydrogels were characterized by Fourier transform infrared spectroscopy (FTIR, Nicolet 6700, Thermo Scientific, Waltham, MA, USA). Samples were prepared by the KBr pellet method and scanned over the range of 4000–400 cm^−1^ at a resolution of 2 cm^−1^ for 64 scans. HA and HA-ADH were dissolved in D_2_O, and their ^1^H NMR spectra were recorded using a nuclear magnetic resonance spectrometer ((Bruker AVANCE III HD 600 MHz, Bruker BioSpin GmbH, Karlsruhe, Germany). The degree of substitution was calculated from the integral ratio of the methyl protons of the HA acetamide group to the characteristic methylene protons of ADH.

The internal morphology and elemental distribution of the hydrogels were characterized by field-emission scanning electron microscopy (FE-SEM, Gemini 300, Carl Zeiss AG, Jena, Germany) coupled with energy-dispersive spectroscopy (EDS). After lyophilization for 48 h, the samples were fractured in liquid nitrogen to expose the cross-sections, sputter-coated with gold, and then subjected to SEM observation and elemental mapping of N and Cu.

#### 3.4.2. Swelling, Degradation, and Antioxidant Properties of Hydrogels

The swelling behavior of the hydrogels was determined by immersion in PBS at 37 °C. Briefly, lyophilized samples were weighed to obtain the initial mass (W_0_), immersed in PBS, and removed at predetermined time points. After gently blotting excess surface water, the samples were weighed again (W_t_), and the swelling ratio was calculated accordingly.

(3) $$\mathrm{Swelling}\;\mathrm{ratio}\;(\%)=\frac{W_{t}-W_{0}}{W_{0}}\times 100\%$$

For the in vitro degradation study, hydrogel samples were incubated in PBS at 37 °C. At predetermined time points, the samples were collected, lyophilized, and weighed to obtain the remaining dry weight (W_2_). The residual weight was calculated using the initial dry weight (W_1_) as the reference.

(4) $$\mathrm{Remaining}\;\mathrm{weight}\;(\%)=\frac{W_{2}}{W_{1}}\times 100\%$$

The antioxidant activity of the hydrogels was evaluated by a DPPH radical scavenging assay with slight modification of a reported method. HA-ADH, HA-Cu, and HA-Cu/Cur were used as the sample groups. Equal volumes of sample solution and 0.04 mg/mL DPPH solution were mixed and incubated in the dark at room temperature for 30 min, and the absorbance was measured at 517 nm. The DPPH radical scavenging rate was then calculated accordingly. All experiments were performed with at least three parallel samples.

(5) $$\mathrm{DPP}H\;\mathrm{radical}\;\mathrm{scavenging}\;\mathrm{rate}\;(\%)\;=(1-\frac{A_{\mathrm{sa}}-A_{\mathrm{sb}}}{A_{\mathrm{co}}-A_{\mathrm{cb}}})\times 100\%$$

#### 3.4.3. PH Responsiveness, Adhesion, Self-Healing, and Rheological Properties of Hydrogels

The pH responsiveness of the hydrogels was evaluated by a visual method. Briefly, hydrogel samples were placed in vials, treated with 0.1 M HCl, and gently shaken. The gel-sol transition process was observed by tilting the vials and recorded photographically.

The adhesive properties of HA-Cu and HA-Cu/Cur were qualitatively assessed by attaching the hydrogels to plastic, glass, metal, and porcine skin surfaces. After gentle pressing, their attachment stability under static, bending, and stretching conditions was observed. For the self-healing test, differently colored HA-Cu and HA-Cu/Cur disk-shaped hydrogels were cut through the center and rejoined at the freshly exposed interfaces. After standing at room temperature for a certain period, the integrity of the interface was examined, and whether cracking or separation occurred under gravity or slight external force was recorded.

The rheological properties of the hydrogels were measured using a rotational rheometer equipped with a 20 mm parallel-plate geometry and a gap of 1 mm. Frequency sweep tests were performed over a range of 0.1–10 Hz at a strain of 1%. Strain sweep tests were conducted from 1% to 1000% at a constant angular frequency of 1 rad/s. Steady shear tests were carried out over a shear rate range of 0.1–100 s^−1^ to measure the apparent viscosity. The storage modulus (G′), loss modulus (G″), and viscosity were recorded to evaluate the viscoelasticity, structural stability, and shear-thinning behavior of the hydrogels.

### 3.5. Antibacterial Activity and Antibacterial Mechanism of Hydrogels

#### 3.5.1. Antibacterial Activity of Hydrogels

*Staphylococcus aureus* (*S. aureus*) and *Escherichia coli* (*E. coli*) were used as model bacterial strains. Frozen stocks were revived in liquid medium and cultured overnight at 37 °C with shaking at 180 rpm. The bacterial suspension was then diluted 1:100 in fresh medium and further cultured to the logarithmic phase (OD_600_ ≈ 0.5), followed by dilution to the required concentration for subsequent experiments.

The in vitro antibacterial activity of the hydrogels was evaluated by the drop plate counting method, inhibition zone assay, and bacterial growth curve analysis. For the drop plate counting assay, the bacterial suspension was adjusted to approximately 1 × 10^6^ CFU/mL and divided into four groups: PBS control, Cur group (15 μM), HA-Cu group, and HA-Cu/Cur group. The bacterial suspension was mixed with each sample at a volume ratio of 1:1 and incubated at 37 °C for 3 h. After incubation, the bacterial suspensions were serially diluted tenfold, and aliquots of each dilution were spotted onto agar plates for colony counting after incubation. For the inhibition zone assay, bacterial suspensions at 1 × 10^6^ CFU/mL were evenly spread onto agar plates, and hydrogel discs with a diameter of 10 mm were placed on the surface of the inoculated agar. After incubation at 37 °C for 16–18 h, the diameters of the inhibition zones were measured. For bacterial growth curve analysis, logarithmic-phase bacterial suspensions were diluted to OD600 ≈ 0.1, added to 96-well plates, and co-incubated with the corresponding samples. The OD600 values were recorded at 0, 2, 4, 6, 8, 12, and 24 h.

#### 3.5.2. Antibacterial Mechanism of Hydrogels

To investigate the antibacterial mechanism of the materials, bacterial cells treated with different samples were collected, washed with PBS, and fixed with 2.5% glutaraldehyde. After graded ethanol dehydration, critical point drying, and sputter coating, the ultrastructural changes on the bacterial surface were observed by SEM.

The anti-biofilm activity of the hydrogels was evaluated using a mature biofilm model combined with crystal violet staining. Briefly, bacterial suspensions of *E*. *coli* or *S. aureus* (1 × 10^6^ CFU/mL) prepared in LB medium were added to sterile polystyrene 24-well culture plates (1 mL per well) and incubated statically at 37 °C for 24 h to allow initial bacterial adhesion. The culture supernatant was then carefully removed and replaced with an equal volume of fresh prewarmed medium, followed by further incubation at 37 °C for an additional 24 h to obtain mature biofilms. After biofilm formation, the culture medium was removed and the wells were gently washed three times with sterile PBS (0.01 M, pH 7.4) to remove non-adherent bacteria. Subsequently, the samples were placed onto the preformed biofilms, and 500 μL sterile PBS was added to each well to maintain a moist environment. After incubation at 37 °C for 3 h, the wells were washed three times with PBS. The remaining biofilms were fixed with methanol for 15 min and stained with 0.1% (*w*/*v*) crystal violet for 20 min. Excess dye was removed by washing with deionized water, and the bound crystal violet was dissolved in 33% (*v*/*v*) acetic acid. Finally, 200 μL of the resulting solution was transferred to a 96-well plate, and the absorbance at 570 nm was measured using a microplate reader to quantify the residual biofilm biomass.

To further explore the antibacterial mechanism, intracellular ATP and ROS levels in bacteria were determined. For ATP measurement, the bacterial suspension was mixed with the samples at a volume ratio of 1:1 and incubated at 37 °C for 3 h. The bacterial cells were then collected by centrifugation, washed with PBS, lysed, and the supernatant was reacted with ATP working solution. Relative luminescence was measured using a chemiluminescence assay, and the ATP content was calculated according to the ATP standard curve. For ROS measurement, bacterial cells were resuspended and incubated with DCFH-DA (final concentration: 10 μM) at 37 °C in the dark for 30 min. After washing to remove excess probe, the cells were further incubated with different samples for 1 h, and the fluorescence intensity was measured to reflect the intracellular ROS level. All experiments were performed with at least three parallel samples.

### 3.6. Cellular Functional Evaluation of Hydrogels

#### 3.6.1. Cell Culture

Mouse fibroblasts (L929) and mouse macrophages (RAW 264.7) were cultured in MEM or DMEM supplemented with 10% fetal bovine serum (FBS), 100 U/mL penicillin, and 100 μg/mL streptomycin at 37 °C in a humidified incubator containing 5% CO_2_.

#### 3.6.2. Hemocompatibility

The hemocompatibility of the materials was evaluated by a hemolysis assay using extracts of HA-Cu and HA-Cu/Cur hydrogels. The hydrogel extracts were prepared at concentrations of 15, 10, and 5 mg/mL and mixed with an equal volume of 4% mouse red blood cell suspension to obtain a final erythrocyte concentration of 2%. Red blood cell suspensions treated with 1% Triton X-100 and PBS were used as the positive and negative controls, respectively. All samples were incubated at 37 °C for 3 h and then centrifuged. The supernatants were collected, and the absorbance was measured at 545 nm. Each treatment was tested in triplicate. The hemolysis ratio was calculated according to the following equation:

(6) $$\mathrm{Hemolysis}\;\mathrm{rate}\;(\%)\;=\frac{{\mathrm{OD}}_{\mathrm{sam}}-{\mathrm{OD}}_{\mathrm{neg}}}{{\mathrm{OD}}_{\mathrm{pos}}-{\mathrm{OD}}_{\mathrm{neg}}}\times 100\%$$

OD_sam_ is the absorbance value of the sample group (red blood cells treated with the material samples), OD_neg_ is the absorbance value of the negative control group (red blood cells treated with PBS), and OD_pos_ is the absorbance value of the positive control group (red blood cells treated with 1% Triton X-100).

#### 3.6.3. Cytocompatibility

Cytocompatibility was evaluated by the MTT assay. L929 and RAW 264.7 cells were seeded in 96-well plates at a density of 1 × 10^4^ cells/well and cultured for 24 h at 37 °C in a humidified atmosphere containing 5% CO_2_. The culture medium was then replaced with medium containing hydrogel extracts at different concentrations or medium containing 15 μM Cur, while complete medium without any material was used as the control. After 24 h of incubation, 10 μL of MTT solution (5 mg/mL) was added to each well and incubated for 4 h. The supernatant was then removed, and 150 μL of DMSO was added to dissolve the formazan crystals. The absorbance was measured at 490 nm. The relative growth rate (RGR) was calculated according to the following equation:

(7) $$\mathrm{Cell}\;\mathrm{viability}\;(\%)\;=\frac{{\mathrm{OD}}_{a}-{\mathrm{OD}}_{b}}{{\mathrm{OD}}_{c}-{\mathrm{OD}}_{b}}\times 100\%$$

OD_a_ is the absorbance value of the experimental group (cells treated with hydrogel extracts or Cur micelles), OD_c_ is the absorbance value of the control group (cells cultured in complete medium only), and OD_b_ is the absorbance value of the blank group (wells containing the MTT reaction system without cells or materials) for background correction.

#### 3.6.4. Transwell Cell Migration Assay

After confirming the cytocompatibility of the materials, a Transwell migration assay was further performed to evaluate their effects on L929 cell migration. In 24-well Transwell inserts, the lower chambers were filled with HA-Cu extract (5 mg/mL), HA-Cu/Cur extract (5 mg/mL, containing 15 μM Cur), Cur solution (15 μM), or complete medium. L929 cells suspended in serum-free medium were seeded into the upper chambers at a density of 1 × 10^5^ cells/well. After incubation for 24 h, the migrated cells were fixed, stained with crystal violet, and photographed under an inverted microscope after removal of the non-migrated cells on the upper surface. Four to five random fields were selected for each sample, and the number of migrated cells was quantified using ImageJ. Image analysis was performed using ImageJ software (version 1.54p, National Institutes of Health, Bethesda, MD, USA).

#### 3.6.5. In Vitro Anti-Inflammatory Activity of Hydrogels

To further evaluate the anti-inflammatory activity of the materials, an LPS-induced inflammatory model was established using RAW 264.7 cells. RAW 264.7 cells were seeded in 6-well plates at a density of 1 × 10^6^ cells/well. After cell attachment, the cells were divided into five groups: Blank, LPS, Cur + LPS, HA-Cu + LPS, and HA-Cu/Cur + LPS. The final concentrations of HA-Cu and HA-Cu/Cur extracts were both 5 mg/mL, and the concentration of Cur was 15 μM. After pretreatment for 2 h, all groups except the Blank group were stimulated with LPS at a final concentration of 1 μg/mL and further incubated for 24 h. The culture supernatants were then collected, and the levels of TNF-α, IL-6, and IL-1β were determined using ELISA kits according to the manufacturers’ instructions to evaluate the regulatory effects of different treatments on inflammatory cytokine secretion. Mouse TNF-α, IL-1β, and IL-6 ELISA kits were purchased from Beyotime Biotechnology Co., Ltd. (Shanghai, China).

#### 3.6.6. Statistics and Analysis

All experiments were performed in triplicate, and the data are presented as mean ± standard deviation (SD). Statistical analyses were conducted using Origin 2021 software (OriginLab Corporation, Northampton, MA, USA). Differences among groups were evaluated using one-way analysis of variance (ANOVA), followed by Fisher’s least significant difference (LSD) test and Duncan’s multiple range test for post hoc multiple comparisons. Different letters indicate statistically significant differences among groups. A value of *p* < 0.05 was considered statistically significant. No formal randomization or blinding procedures were applied in this study, as the majority of experimental outcomes were based on objective quantitative measurements.

## 4. Conclusions

In this study, a multifunctional HA-Cu/Cur hydrogel was successfully developed through dynamic Cu^2+^–hydrazide coordination and the incorporation of curcumin-loaded micelles. The proposed design integrates the antibacterial activity of Cu^2+^ with the anti-inflammatory and antioxidant properties of curcumin within a single hyaluronic acid-based platform, enabling simultaneous modulation of bacterial infection and inflammatory responses. Mechanistically, the hydrogel exhibited pronounced antibacterial activity against both *E. coli* and *S. aureus*, which was associated with bacterial membrane disruption, ATP depletion, and elevated intracellular ROS levels, while the incorporation of curcumin further enhanced biofilm removal and anti-inflammatory performance without compromising antibacterial efficacy. Compared with the HA-Cu hydrogel, HA-Cu/Cur demonstrated superior biofilm eradication and more effective suppression of pro-inflammatory cytokines, highlighting the benefit of combining Cu^2+^-mediated antibacterial activity with curcumin-mediated microenvironment regulation.

Nevertheless, several limitations should be acknowledged. The present study was limited to in vitro evaluations, and the relationship between the pH-responsive behavior of the hydrogel and the biological activities of the incorporated components was not further investigated. Therefore, the in vivo wound-healing efficacy, biosafety, and long-term therapeutic performance of the hydrogel remain to be validated. Future studies will focus on animal wound models and more detailed mechanistic investigations to further assess the translational potential of this multifunctional hydrogel system.

Overall, the HA-Cu/Cur hydrogel represents a promising strategy for the development of multifunctional wound dressings capable of simultaneously combating bacterial infection and regulating the inflammatory microenvironment.

## Data Availability

The original contributions presented in this study are included in the article. Further inquiries can be directed to the corresponding authors.

## Abbreviations

ADH	Adipic Dihydrazide
DCFH-DA	2′,7′-Dichlorodihydrofluorescein Diacetate
DLS	Dynamic Light Scattering
DMSO	Dimethyl sulfoxide
DPPH	1,1-Diphenyl-2-Picrylhydrazyl Radical 2,2-Diphenyl-1-(2,4,6-Trinitrophenyl) Hydrazyl
*E. coli*	*Escherichia coli*
ECM	Extracellular matrix
EDC·HCl	N-(3-Dimethylaminopropyl)-N’-Ethylcarbodiimide Hydrochloride
FTIR	Fourier transform infrared spectroscopy
HA	Hyaluronic Acid
HA-ADH	Hyaluronic Acid–Adipic Dihydrazide
NHS	N-Hydroxy Succinimide
NMR	Nuclear magnetic resonance
PBS	Phosphate-buffered saline
ROS	Reactive oxygen species
*S. aureus*	*Staphylococcus aureus*
SEM	Scanning electron microscope
